# Real-Time Traffic Risk Detection Model Using Smart Mobile Device

**DOI:** 10.3390/s18113686

**Published:** 2018-10-30

**Authors:** Soyoung Park, Homin Han, Byeong-Su Kim, Jun-Ho Noh, Jeonghee Chi, Mi-Jung Choi

**Affiliations:** 1 Department of Software, Konkuk University, Seoul 05029, Korea; soyoungpark@konkuk.ac.kr (S.P.); hi_test@naver.com (H.H.); point91@konkuk.ac.kr (B.-S.K.); lf_pury@naver.com (J.-H.N.); jhchi@konkuk.ac.kr (J.C.); 2 Department of Computer Science, Kangwon National University, Gangwon-do 24341, Korea

**Keywords:** traffic risk detection, real-time service, machine learning, deceleration pattern, smart mobile device

## Abstract

Automatically recognizing dangerous situations for a vehicle and quickly sharing this information with nearby vehicles is the most essential technology for road safety. In this paper, we propose a real-time deceleration pattern-based traffic risk detection system using smart mobile devices. Our system detects a dangerous situation through machine learning on the deceleration patterns of a driver by considering the vehicle’s headway distance. In order to estimate the vehicle’s headway distance, we introduce a practical vehicle detection method that exploits the shadows on the road and the taillights of the vehicle. For deceleration pattern analysis, the proposed system leverages three machine learning models: neural network, random forest, and clustering. Based on these learning models, we propose two types of decision models to make the final decisions on dangerous situations, and suggest three types of improvements to continuously enhance the traffic risk detection model. Finally, we analyze the accuracy of the proposed model based on actual driving data collected by driving on Seoul city roadways and the Gyeongbu expressway. We also propose an optimal solution for traffic risk detection by analyzing the performance between the proposed decision models and the improvement techniques.

## 1. Introduction

Advanced driver assistance systems for driver safety and convenience are growing rapidly and are widely used in the vehicular domain [1]. In particular, various smart phone-based driving assistance systems, including the detection of front vehicles and obstacles, the headway distance, and time to a collision estimation [2,3,4]; the recognition of various driver behaviors and road conditions [5,6,7,8]; the identification of driving styles [9,10,11,12], the detection of emergent braking [13], and an accident alert for preventing a secondary accident [14,15] have been actively proposed. The smart phone-based driving assistant systems are still attractive, since they can be directly used for ordinary vehicles without additional sensor equipment for monitoring driving situations. In this paper, we introduce a smart phone-based driver assistance system that can detect a dangerous situation by machine learning on emergent braking.

Emergent braking or stopping on the road is very dangerous, because it can cause a collision with the following cars. Without alerts on the emergent situation to nearby vehicles, a secondary collision can occur. Thus, a real-time detection of dangerous situations that can be shared with nearby vehicles is a very essential technique for road safety. Our study focuses on the dangerous situation detection based on the dangerous deceleration that can lead to accidents. Since emergent braking is inevitable in all risk situations, the occurrence of emergent braking can be used as a basic indicator of a risk situation. However, depending on the driving style of a driver, non-hazardous and habitual sudden braking may occur frequently as well. If a warning message for every case of simple emergent braking, which is not harmful to surrounding vehicles, is produced and shared with neighboring vehicles, then it will become excessive noise to nearby vehicles. Therefore, it is necessary to detect only dangerous braking that is threatening to nearby vehicles and should be propagated to them. At this point, the vehicle’s headway distance at the moment of sudden braking is also considered in judging the dangerous braking.

Consequently, we propose a practical traffic risk detection model that automatically detects dangerous situations by monitoring and analyzing a driver’s deceleration pattern, including the vehicle’s headway distance in real time. Our model obtains essential driving information, such as the headway distance to the front vehicle, driving speed, and moving distance using the global positioning satellite (GPS) sensor and camera of the smart mobile device. 

The proposed system consists of four modules: user interface, driving data management, risk detection, and communication. We focus in this paper on the driving data management and the risk detection, as they are the core concepts of this research. The driving data management module continuously collects driving information, including the vehicle’s headway distance, and extracts deceleration segments from the data. The risk detection module is divided into a learning module and a decision module. The learning module performs machine learning on dangerous deceleration based on three learning algorithms: neural network, random forest, and clustering. Since both supervised learning (neural network and random forest) and unsupervised learning (clustering) are used in parallel in the learning module, the accuracy of the risk situation detection will be enhanced. The decision module makes the final decision on every single deceleration segment based on the results of the learning module. The decision module outputs one of “danger”, “suspicious”, or “general”. We suggest two types of decision algorithms for the final decision, and compare the performances of both algorithms. In addition, it constructs new training data from the clearly classified deceleration segments. The new training set is periodically sent back to the learning module so as to continuously enhance the learning module. We also propose three types of enhancement algorithms to improve the learning module. The performance will be also analyzed in Section 4.

The contributions of our work are as follows. (1) We develop a smart mobile device-based real-time traffic risk detection model that monitors driving behavior and detects dangerous emergent braking. (2) We provide a practical vehicle detection and headway distance estimation method. (3) We use real driving data collected from two drivers for three months to build risk deceleration patterns. (4) Our traffic risk detection model uses various machine learning algorithms on these risk deceleration patterns to improve the detection accuracy. Finally, we evaluate the performance of the proposed model, including the accuracy of the risk situation detection, for various decisions and enhancement algorithms with real traffic data gathered by driving on Seoul city roads and the Gyeongbu expressway. 

The rest of this paper is as follows. In Section 2, we briefly mention related works; then, we describe our proposed risk detection model in detail in Section 3. We analyze the simulated performance in Section 4, and conclude the paper in Section 5.

## 2. Related Work

Despite the amount of research on ITS (intelligent transportation systems) and ADAS (advanced driving-assistance systems) for road safety over the past decade, traffic accidents remain the eighth leading cause of death globally, such that 1.25 million people die each year on the world’s roads [16]. Thus, from the perspective that traffic accidents are associated with drivers’ driving styles, studies on building a driver profile, driving behaviors, and driving style recognition have been actively carried out. 

First, currently available driving assistance applications include iOnRoad, Movon FCW, and so on. iOnRoad [2] detects the front vehicle and provides either the headway distance or time to collision to the detected vehicle using a smart phone. Movon FCW [3] detects incoming cars, or cars that are not in full view, and provides actual distance information. Related to obstacle detection, Danescu et al. [4] have proposed a technique for generic, shape-independent real-time obstacle detection for mobile devices. 

Second, technologies for recognizing various driver behaviors and external road conditions [5,6,7], such as left turns, right turns, braking, potholes and bumps, etc., have been proposed using various smart mobile device sensors, including accelerometers, microphones, and GPS sensors. A patent [8] has even proposed a technology that collects driving data using a smart phone and assesses distracted driving, and then provides a driving safety score to the driver and certain related institutes, such as one’s insurance company. 

Related to identifying driving styles, Meiring and Myburgh [9] proposed a method for unique driver identification through driving style analysis using an artificial intelligence algorithm based on a fuzzy logic inference system, hidden Markov models, and support vector machines. Ly, Martin, and Trivedi [10] introduced a scheme that builds a profile of a driver using a vehicle’s inertial sensors from CAN (Controller Area Network)-bus, and provides proper feedback to the driver regarding dangerous driving behavior. Another vehicle status monitoring scheme using traffic data collection and assessment devices equipped in a vehicle was proposed in the patent of Lavie et al. [11]. This scheme also analyzes driving behavior patterns and provides feedback to the driver for the vehicle status. Deng et al. [12] proposed a scheme to classify driving styles into one of three styles (aggressive, moderate, or mild) by analyzing driver braking characteristics using a hidden Markov model. 

Lastly, related to dangerous situation recognition based on emergent braking, Xiao and Liu [13] proposed a mechanism that detects emergent braking through machine learning on brake pedal speed using a probability neural network. Lee, Park, and Yoo [15] suggested a system that detects dangerous emergent braking on expressways using the accelerometer of a smart phone and sends relevant warning messages to nearby vehicles. This study is the most similar to our work of the prior studies, but the main differences are that the dangerous situation detection in the previous scheme [15] depends on the accelerometer of the smart phone, and that the maximum acceleration of 10 m/s^2^ or more is always considered to be dangerous braking.

## 3. Proposed Real-Time Traffic Risk Detection System

### 3.1. System Configuration and Operation

**Figure 1:**
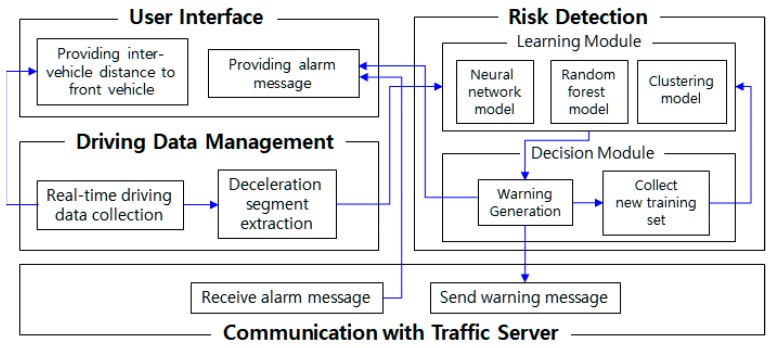
System configuration.

Our system consists of a traffic data management server, vehicles, a smart mobile device equipped with a camera and GPS, and our proposed traffic risk detection application. The proposed traffic risk detection application consists of a user interface module, a driving data management module, a risk detection module, and a communication module with the traffic server. The application basically monitors driving behavior by collecting current GPS position and the distance to the front vehicle every second. After estimating the current speed and travel distance, it extracts deceleration segments in real time. Based on the deceleration segments, the learning module performs machine learning regarding normal deceleration and risk deceleration. The decision module detects risk deceleration based on the learning module for new deceleration segments, and generates a warning message when a deceleration segment is considered dangerous. In such a case, the communication module sends the warning message to the traffic server. The traffic server will then feed alarm signals back to the vehicles surrounding the source vehicle in order to prevent a potential secondary accident caused by the following vehicles. Here, the way in which the traffic server selects only vehicles near the source vehicle is beyond the scope of this paper, so we will leave it for our future work. The system illustration of our proposed model is shown in Figure 1. 

Since it is difficult to define a dangerous deceleration pattern in one sentence, and because such a pattern can occur in various forms depending on the driving situation, we experimentally collected driving data that the driver determined to be dangerous, and used it for machine learning to develop the dangerous pattern in our learning module. Therefore, the neural network, which is a representative labeled-learning method, and the random forest technique, which is suitable for various kinds of classification, are used in parallel. Moreover, since it is necessary to be able to recognize untrained dangerous situations, a clustering technique, which is a representative non-guidance learning model, is used together with these as well. As a result, three learning models are used for our risk deceleration learning.

After establishing an initial risk detection model with the experimentally obtained training data, the decision module determines one of “danger”, “suspicious”, and “normal” for new deceleration data provided in real time. 

Finally, as the driver’s real-time driving data is continuously updated, the risk situation detection model must also be continuously updated. Some deceleration segments that are clearly detected as “dangerous” or “normal” are used to train our detection model periodically. Our detection model can thereby be continuously updated and enhanced to a driver-customized model. 

### 3.2. Driving Data Management

We will describe the data management module and the risk detection module of the proposed system in further detail in this section. Our proposed vehicle detection and the headway distance estimation will be explained first, and the driving data collection and deceleration segment extraction will then be described in detail.

#### 3.2.1. The Front Vehicle Detection and the Inter-Vehicle Distance Estimation

We basically assume that the smart phone is fixed on the dashboard and the forward image of the vehicle is given as an input. The size of the input image is 960 × 540 in consideration of the screen aspect ratio (16:9) of a general smart mobile device and image processing performance. Our mobile application records 60 frames per second and carries out the vehicle detection and the headway distance estimation every two frames. It is assumed that the taillights of the vehicle are in the red color, and are always located at the rear left and right ends of the vehicle.

Due to the unique characteristics of taillights—that the taillights are commonly in red in all vehicles, and are symmetrically located at both ends of the vehicle—our scheme detects the front vehicle and measures the width of the front vehicle by detecting the taillights of the front vehicle. For an efficient taillight search, an ROI (region of interest) designation is performed first. Since the ROI must include the front vehicle, the ROI is determined based on the shadow of the vehicle. The shadow of the vehicle always appears on the road surface, it is contacted with the wheels of the car, the color is darker than the road surface, and it has a width greater than the width of the car. So, the minimum area of interest, which can contain the whole vehicle based on the shadow, can be determined. Once the ROI is determined, the taillights of the vehicle are extracted in the ROI, and the width of vehicle can be measured by the distance between the taillights. Finally, the headway distance can be measured by the estimated vehicle width and the angle of view of the camera. The proposed scheme consists of five steps: (1) road area extraction by inverse perspective mapping (IPM) [17], (2) car shadow detection, (3) ROI extraction, (4) taillights detection, and (5) inter-vehicle distance estimation. We will describe the detailed algorithms for each stage in next subsections. 

A. Road Area Extraction by IPM (Inverse Perspective Mapping) 

**Figure 2:**
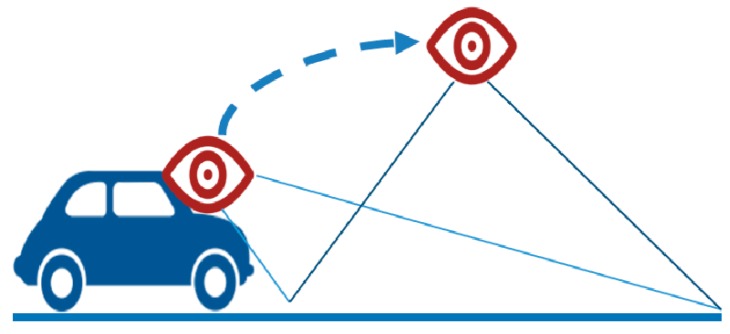
Inverse perspective mapping (IPM) mapping principle.

First, the reduced road area is extracted by applying IPM as a predecessor work for extracting shadows from the road surface. IPM is a method of generating the result of removing the perspective effect by moving the point of the camera to the position vertically above the road, as shown in Figure 2. 

**Figure 3:**
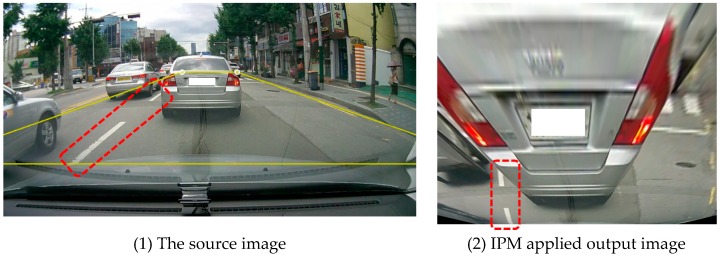
Removal of the perspective effect by IPM.

The image provided by the mobile application shows the road surface at the bottom of the center of the image, as shown in Figure 3-(1), except that the distance between the vehicle and the front vehicle is very close. By applying IPM to the road area, the image as shown in Figure 3-(2), which is similar to being captured from the position vertically top from the road, and in which the background area of the image has been removed, can be obtained. The length of the shadow region is relatively different depending on the distance between the vehicle and the front vehicle. This reduces the relative distance difference of the shadow region, so the length of the shadow has a similar length. 

The Algorithm 1 for applying IPM is given below:

**Algorithm 1:** IPM Transformation//*src*: road area in input image //*dst*: IPM applied output image function **IPM**(*src*, *dst*) *T* = getPerspectiveTransform(*src.coordiate*, *dst.coordinate*) for *x* = 0 to *dst.width*, *y* = 0 to *dst.height* *u* = *T*(0, 0) * *x* + *T*(0, 1) * *y* + *T*(0, 2)    *v* = *T*(1, 0) * *x* + *T*(1, 1) * *y* + *T*(1, 2)    *s* = *T*(2, 0) * *x* + *T*(2, 1) * *y* + *T*(2, 2) *Map_X*(*x*) = *u*/*s* *Map_Y*(*x*) = *v*/*s*  return remap(*src*, *dst*, *Map_X*, *Map_Y*)

The function of getPerspectiveTransform() finds a 3 × 3 perspective transform matrix to transform the coordinates of *src* to the coordinates of *dst*. Suppose that *src.coordinate* is {$(x_{0},y_{0}),\;(x_{1},y_{1}),\;\ldots$} and *dst.coordinate* is {$({x^{\prime }}_{0},{y^{\prime }}_{0}),\;({x^{\prime }}_{1},{y^{\prime }}_{1}),\;\ldots$}. The perspective transform matrix *T* satisfies the following equation:

(1) $$\left[\begin{array}{c} t_{i}{x^{\prime }}_{i}\\ t_{i}{y^{\prime }}_{i}\\ t_{i} \end{array}\right]=T\cdot \left[\begin{array}{c} x_{i}\\ y_{i}\\ 1 \end{array}\right]$$

The function of remap() performs geometric transformation on the image by the mapping function *Map_X* and *Map_Y*. That is, *dst*(*x*, *y*) is determined as *src*(*Map_X*(*x*, *y*), *Map_Y*(*x*, *y*)) by the remap(*src*, *dst*, *Map_X*, *Map_Y*). The iteration part of the function finds the mapping function based on the perspective transform matrix. Finally, the IPM-applied *dst* can be obtained. 

B. Car Shadow Detection

**Figure 4:**
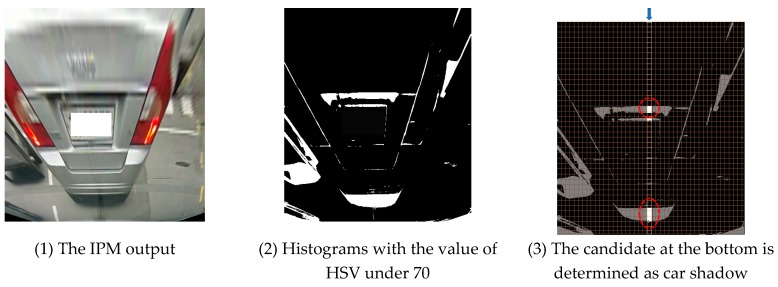
Car shadow detection with color histogram.

The shadow of the car can be extracted from the IPM-applied image using the color histogram. First, in the HSV (Hue, Saturation, Value) color space, only the area whose brightness (value: 0~255) is 70 or less is classified, as shown in Figure 4-(2). At this point, other objects besides the car shadow can be recognized as shadows, too. In order to find the car shadow, the following characteristics of the car shadows are used: (1) the front car is in the center of the road, (2) shadow appears at the bottom, and (3) there is no obstacle between the front car and the traveling car. Based on the features, the area that is positioned at the center bottom of the image is determined as the car shadow. Consequently, the candidate at the bottom between the two red circled candidates in Figure 4-(3) is determined as the car shadow. 

By applying the mapping functions used for IPM image generation inversely, the shadow area coordinates on the original image are acquired. 

C. ROI (Region of Interest) Configuration

**Figure 5:**
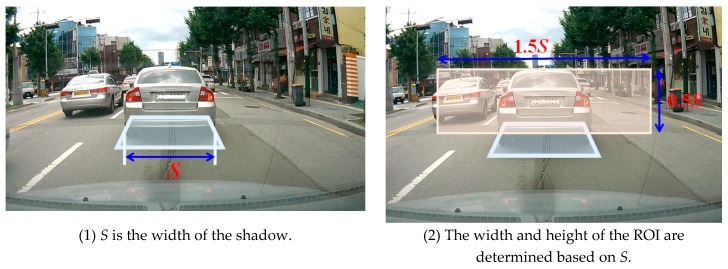
Region of interest (ROI) setting when car shadow is detected.

Once the car shadow area has been detected, the ROI area for detecting the taillights of the vehicle is configured. The ROI is defined as the minimum square area, which includes the entire front car in the center of the area. Since the width of the shadow is always similar to the width of the car, the ROI can be determined based on the width of the shadow. Let the width of the shadow be *S*. The width of ROI is set as 1.5*S*, and the height of ROI is set as 0.5*S*. Finally, the ROI, as shown in Figure 5, is defined as a square with the previously defined width and height based on the top of the shadow. 

**Figure 6:**
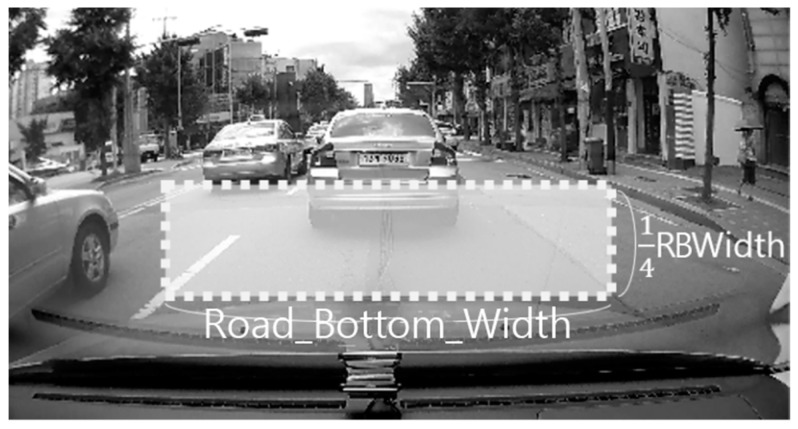
Predefined ROI when failing to detect the car shadow.

However, the extraction of the shadow area can fail either if the headway distance is too close, so that the shadow of the vehicle has been hidden by the current vehicle, or if there is no vehicle in front of the current vehicle. In the case of failing to extract the shadow area, a predefined ROI area is used. As shown in Figure 6, the ROI is determined by the systemically predefined road bottom width. 

The Algorithm 2 to configure the ROI is given below:

**Algorithm 2:** ROI Configuration// *img*: a source image // *WIDTH*: the width of the image, 960 px // *HEIGHT*: the height of the image, 540 px // *Road_Top*: a predefined top position of the road area in the image, HEIGHT*2.7/10 // *Road_Bottom_Width*: a predefined width of the bottom side of the road area, *WIDTH*/3*2 // *Road_Height*: a predefined height of the road area, HEIGHT*4.3/10 function **getROI(***img***)** *black_area* = getBlackArea(*img*) if *black_area*! = False *S* = *black_area.right*−*black_area.left* *x_coord* = (*WIDTH*–1.5*S)/2 *y_coord* = *black_area.top*–0.5*S *ROI*(*x*,*y*,*w*,*h*) = (*x_coord*, *y_coord*, 1.5*S, 0.5*S) else *S* = *Road_Bottom_Width*   *x_coord* = (*WIDTH*–1.5*S)/2   *y_coord* = *Road_Top*+*Road_Height*−0.5*S *ROI*(*x*,*y*,*w*,*h*) = (*x_coord*, *y_coord*, 1.5*S, 0.5*S) return *ROI*

D. Taillights Detection

After configuring the ROI area, the detection of taillights is performed. The taillights of the vehicle have common features, such as: (1) they are always symmetrically positioned at the rear left and right ends of the vehicle, and (2) they are all in red color. Thus, if all the following five conditions are satisfied, it is detected as a pair of taillights.

(a) For the HSV color space, the hue value (hue: 0°~180°) is less than or equal to 10° or greater than or equal to 170°.
(b) They are located symmetrically in the left and right area from the center of the ROI, respectively.
(c) The size of red area is bigger than the predefined threshold value *α*
(d) For the two symmetric red areas, the height difference is less than *δ*, and the slope difference is less than *ε.*
(e) The shape of the two selected areas is almost the same and left–right symmetric.

**Figure 7:**
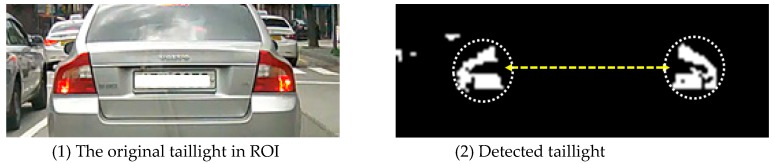
Taillight detection in ROI.

Figure 7 shows the taillight area detected by our algorithms.

As shown in Figure 7-(2), every red area in the ROI is regarded as a taillight candidate. For every candidate, morphological dilation and erosion have been performed to remove noise first, and then, the ROI is split into the left area and the right area by the center of the ROI. For every candidate, the steps from (b) to (e) are repeatedly performed. Suppose a candidate in the left area as *red_left*, and another candidate in the right area as *red_right*. Our algorithm decides if both are the closest to the center of the ROI, if the size of each is bigger than *α*, if the slope difference between two candidates is less than *ε*, and if the height difference between them is less than *δ*. Finally, the test for the shape similarity and symmetricity is carried out.

**Figure 8:**
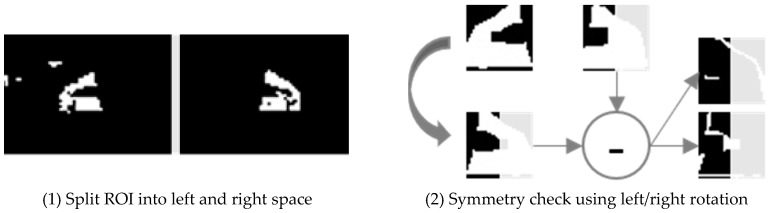
Taillight shape similarity test.

Suppose the left–right rotated result of *red_left* as *red_left*’, as shown in Figure 8-(2). The test simply computes the difference between *red_left*’ and *red_right*. The difference can be computed either by (*red_left*’ − *red_right*) or by (*red_right* − *red_left*’). The smallest difference among them is adopted as the difference. If the size of the difference is less than or equal to 1/5 of *red_left*, then both candidates are determined as the same and symmetric. 

Consequently, a pair of *red_left* and *red_right* is detected as taillights. 

Here, since a red-colored car satisfies our proposed conditions, an exceptional processing for the red-colored car is required. If the size of candidate is larger than 1/5 of the size of ROI, such candidates are excluded from taillight candidates. 

E. Inter-vehicle Distance Estimation

**Figure 9:**
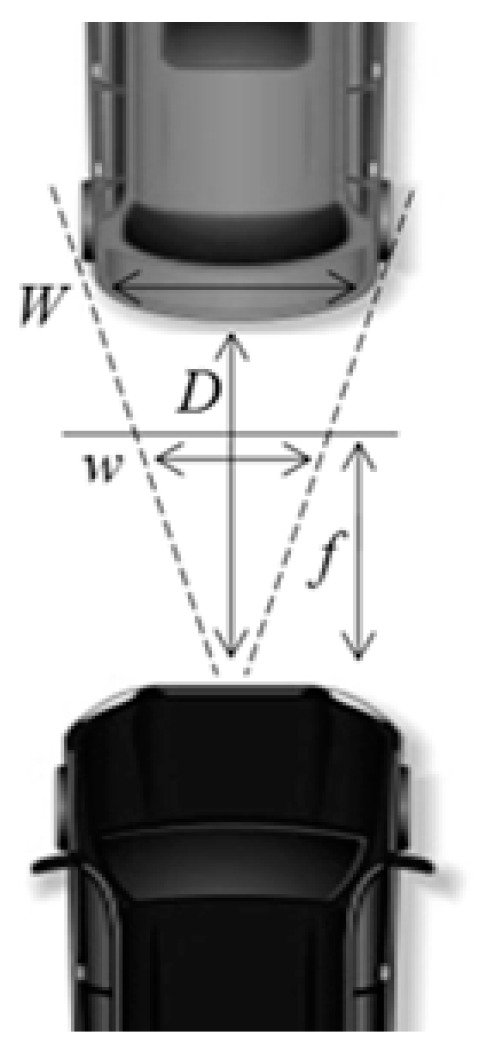
Distance estimation.

After taillight detection has been accomplished, the headway distance estimation to the front vehicle is performed. Since taillights are positioned at the left and right end of the vehicle, the width between taillights represents the width of the vehicle. As shown in Figure 9, suppose that the height of the image is *h*, the angle of view of the camera is *Θ*, the estimated car width is ω, the focal length of the camera is *f*, and the real width of the front car is *W*. Since the real width of the front car is different depending on the type of cars, our algorithm uses an average width (about 1.8 m) of a car for the value of *W*. As a result, the headway distance D is measured by the following Equations (2) and (3).

(2) $$f=\frac{h}{2tan\theta }$$

(3) $$D=\frac{f\cdot W}{\omega }$$

#### 3.2.2. Driving Data Collection 

The main driving data collected by a smart device consists of the current speed, mileage per second, and the headway distance to the front car. The current speed is measured as the travel distance for one second, based on the GPS position. Sathyanarayana et al. [18] showed that the current speed estimation using the GPS of a smart mobile device is as accurate as the speed value obtained from the CAN-bus of a car. The inter-vehicle distance is estimated from the images captured by the camera. The mileage per second is the Euclidean distance between the previous GPS position and the current GPS position. We denote the driving data at time *T* = *i* as *D_i_* = <*V_i_*, *MD_i_*, *VD_i_*>. *V_i_* is the current velocity (km/h), *MD_i_* is the mileage per second (meter), and *VD_i_* represents the inter-vehicle distance (meter).

#### 3.2.3. Deceleration Segment Extraction and Feature Vector Generation

The next step is to extract the deceleration section from the driving data in seconds. A deceleration segment, which is denoted as *DS*, is a subset of sequential driving data. The initial value of *DS* is ∅. For *T* = *i*, *DS* is updated as follows:

$$\begin{array}{l} \mathrm{if}\;(V_{i}\leq V_{i-1})\;\mathrm{then}\;DS=DS\cup \{D_{i}\};\\ \mathrm{else}\;\{\\ \;\mathrm{if}\;|DS|>1\;\mathrm{then}\;\mathrm{output}\;DS;\\ \;\mathrm{reset}\;DS=\{D_{i}\};\\ \;\} \end{array}$$

For example, suppose that *DS* at *T* = *i* is *DS* = {*D_j_*, *D_j_*_+1_, …., *D_i_*_(=*j+k*)_}. That is, *D_j+k_* = *D_i_*. For each *DS* output, a feature vector *x*_i_ for training the learning module is computed. *x_i_* consists of seven attributes: the beginning speed of *DS*, the final speed of *DS*, the average deceleration speed, the maximal deceleration speed, the total travel distance, the total travel time, and the end inter-vehicle distance. Finally, *x_i_* is denoted as *x_i_* = <*V_j_*, *V_i_*, *Avg_i_*, *Min_i_*, *TDist_i_*, *TLen_i_*, *VD_i_*>, and each attribute value can be computed by the following Equations:

(4) $$Diff\left(j\right)=V_{j}-V_{j-1}$$

(5) $$Avg_{i}=\frac{\sum_{l=j+1}^{i}Diff\left(l\right)}{k}$$

(6) $$\underset{l=j+1,\ldots ,i}{Min_{i}=\min}\left\{Diff\left(l\right)\right\}$$

(7) $$TDist_{i}=\sum_{l=j+1}^{i}MD_{l}$$

(8) $$TLen_{i}=k$$

Since the scale of each value in the vector differs from the others, it is finally normalized as having an average of 0 and a variation of 1.

### 3.3. Traffic Risk Detection 

#### 3.3.1. Learning Module

**Figure 10:**
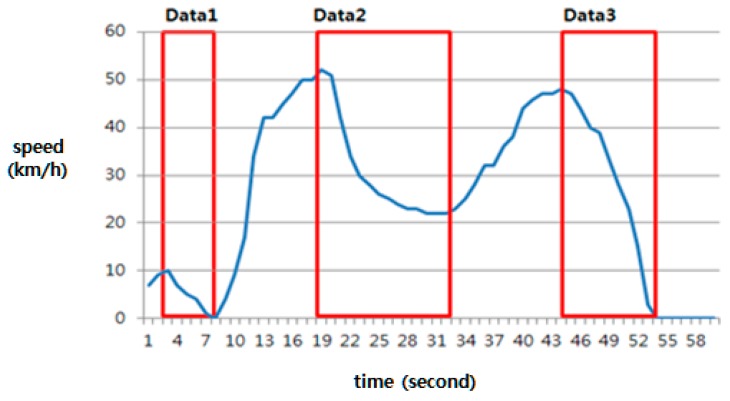
Various deceleration patterns, including sudden braking.

There are various types of deceleration patterns, depending on the traffic conditions and driving style. The following Figure 10 shows three different types of deceleration patterns labeled Data 1, Data 2, and Data 3. All of the patterns include emergent braking. The deceleration labeled Data 1 occurred at low speed, the pattern labeled Data 2 includes sudden deceleration at the beginning, but slow deceleration around 30 km/h afterward, and the pattern of Data 3 represents risk deceleration, in which the driver felt real danger. Therefore, a technique is needed that distinguishes only the pattern of Data 3 as dangerous.

In this study, we use machine learning technology to classify the risk situations that drivers perceive as dangerous. Since the proposed model should be able to be executed in real time on a smart mobile device, instead of using the deep learning technique, we use a combination of various basic machine learning techniques. For the labeled learning, we used the neural network model, which is the most typical type of labeled learning, and the random forest method, which has excellent classification performance in various situations. In addition, the *k*-nearest neighboring method was used as the non-labeled learning model. 

A. Initial Training and Test Data Collection 

First, it is necessary to obtain training data that can clearly judge whether braking is dangerous or normal. In order to achieve this, we have gathered real deceleration data that two drivers both empirically feel to be dangerous. The two drivers gathered actual driving data using our mobile application on their commutes, which included both city roads and highways. We let drivers touch the mobile device whenever they felt dangerous deceleration, and such data was collected as risk training data. Since risk deceleration does not occur often, the amount of training data was insufficient. Thus, additional experimental data, which have similar patterns to the risk training data, have been added to the training data in order to increase the reliability of our risk detection model. Details of the experimental data are described in Section 4. 

B. Neural Network Module 

The first machine learning model is the neural network model. We have used a multi-layer perceptron (MLP) model with two hidden layers, each of which has three nodes. It finally outputs two class labels, such that 1 means “dangerous” and 0 means “normal”. For a given train set *X* = {(*x*_1_, *c*_1_), (*x*_2_, *c*_2_), …, (*x_N_*, *c_N_*)}, where *x_i_* is the *i*-th feature vector and *c_i_* is the class label ∈{0,1}, the MLP learning function for each *x_t_* ∈ *X* is defined as follows: 

the *k*-th node at the *j*-th layer for 1 ≤ *j* ≤3:

(9) $$O_{k}=g(\sum_{i=1}^{p}Z_{i}^{j-1}W_{ki}^{j-1}+b_{k}^{j}),$$

where $Z_{i}^{j-1}$ is the *i*-th value at the (*j*−1)-th layer,

$W_{ki}^{j-1}$ is the weight at the (*j*−1)-th layer, and

$b_{k}^{j}$ is an the bias added to the *j*-th layer.

*k* is the number of nodes at the *j*-th layer and *p* is the number of nodes at the (*j*−1)-th layer. *j* = 1 represents the first hidden layer, and *j* = 3 is the output layer. If *j* = 1, then $Z_{i}^{j-1}=x_{i}^{t}$, where $x_{i}^{t}$ is the *i*-th value in *x_t_*. *g*(*x*) is the activation function. The following hyperbolic tangent function has been used as the activation function:

(10) $$g\left(x\right)=\frac{e^{x}-e^{-x}}{e^{x}+e^{-x}}$$

For the *k*-th training sample *x_k_*, let the estimated output of *x_k_* at the output layer be *O_k_*. The output is classified into *y_k_* by the following softmax function, as in Equation (11). In addition, the error rate (*E_N_*) between every pair of *y_k_* and *c_k_* is computed by the cross-entropy function, as in Equation (12):

(11) $$y_{k}=Softmax\left(o_{k}\right)=\frac{e^{o_{k}}}{\sum_{i=1}^{2}e^{o_{i}}}$$

(12) $$E_{N}=-c_{k}\log_{2}\left(y_{k}\right)-\left(1-c_{k}\right)\log_{2}\left(1-y_{k}\right)$$

Consequently, the model minimizes the error rate by repeatedly updating the weights *W*_1_ and *W*_2_. The error back-propagation algorithm based on the SGD (stochastic gradient descent) from the output layer to the previous layer is used to minimize the error rate.

C. Random Forest Module 

Secondly, the random forest model is used to protect the risk detection model from being overfit to the training set. Since the size of the training set is not big, the number of decision trees is set to 50. Each tree has two outputs so as to match the previous neural network model. Random trees are initially established by a randomly chosen training set. The tree node split for constructing a decision tree is also performed by randomly chosen attributes. The number of randomly selected attributes for the tree node split, which is denoted as *rand_features*, has a significant impact on the performance of the random forest model. Thus, *rand_feature* is determined by the following Equation (13), as proposed by Breiman [19]. *n* means the total number of attributes:

(13) $$rand\_features=int\left(\log_{2}n+1\right)$$

Each node of a decision tree is split into a binary tree. Let *RF* = {*f*_1_, *f*_2_, …, *f_l_*} be a subset of randomly selected features. For each *f_i_*, it finds a reference value among all the values of *f_i_* that minimizes the entropy between the reference value and all of the other values. Suppose that a selected reference value for *f_i_* is *v_i_*. For a given training sample data, the entropy by *v_i_* is determined by the following entropy function *E_R_*:

*L* = a subset of data that has values lower than *v_i_*;
*U* = a subset of data that has values equal or greater than *v_i_*;
$eL=-(P(class=0|L)\log_{2}P\left(class=0|L\right)+P(class=1|L)\log_{2}P\left(class=1|L\right))$;
$eU=-(P(class=0|U)\log_{2}P\left(class=0|U\right)+P(class=1|U)\log_{2}P\left(class=1|U\right))$;
  (14) $$E_{R}=\frac{eL\cdot \left|L\right|+eU\cdot \left|U\right|}{\left|L\right|+\left|U\right|}$$

The node is finally split by the feature that has the smallest entropy. The tree node split is also repeated until no further split occurs. Once a random forest has been established, the final output is determined as the most frequent decision of the decision trees. 

D. Clustering Module 

In order to make the proposed model work effectively even for untrained risk situations, a clustering model, which is a typical unsupervised learning method, is used as well. Our training dataset consists of seven attributes. In order to increase the accuracy of clustering, the two main attributes that have the greatest effect on the classification are extracted, and the clustering is performed based on the principal attributes. The principal components were extracted from the histogram analysis of the “dangerous” and “normal” conditions for each attribute. We have simply compared the difference in average values between both groups for each attribute; the maximum deceleration has about 47% difference, the average deceleration has about 29% difference, the inter-vehicle distance has about 18% difference, the end speed has about 12% difference, and the remainders have about 5% difference. Thus, the “maximum deceleration” and “average deceleration” were extracted as the main components. Finally, a *k*NN (*k* Nearest Neighbors) algorithm is used for the classification based on the main components for the following two reasons. (1) We have the training dataset that has been already classified. (2) The performance of *k*-means clustering, which is another representative clustering algorithm, deeply depends on the initial cluster heads that are randomly selected. The number of *k* neighbors is set to 5. The Euclidean distance has been used to compute the distance between two points. For input data, classification is computed from a simple majority vote of the nearest neighbors of each input point: the data class that has the most representatives within the nearest neighbors of the input point is assigned to the input data. 

In this way, the initial learning module based on the three different machine learning algorithms is established.

#### 3.3.2. Decision Module

A. Making the Final Decision

Based on the initial learning module, the decision module makes the final decision on new driving data in real-time. The decision classifies braking as either “danger”, “suspicious”, or “normal”. During driving, the driving data management module extracts deceleration segments, and every deceleration segment is given to the decision module. We have experimented with two decision algorithms named *DM_all_* and *DM*_2_ in this study. For each deceleration segment, *DM_all_* works as follows:

If all models decide as danger, the final decision is “danger”,
If all models decide as normal, the final decision is “normal”,
otherwise, the final decision is “suspicious”.

In contrast, *DM*_2_ works as follows:

If two or more models decide as danger, the final decision is “danger”,
If all models decide as normal, the final decision is “normal”,
otherwise, the final decision is “suspicious”.

From the conclusion, it is experimentally proven that the performance of *DM*_2_ is better than that of *DM_all_*, so *DM*_2_ has been adopted as our main decision rule. The performance analysis of the two methods will be described in detail in Section 4. Finally, a warning message is transmitted to the traffic server if and only if the final decision is “dangerous”.

B. Enhancing the Risk-Detecting Model 

The deceleration data through which final decisions have been made are used as new training data for updating and enhancing the risk situation detection model. As driver-specific deceleration data is continually added to the common initial risk-detection model, the reliability of the initial model can be continuously enhanced, and the model is updated to a driver-customized model. Three types of enhancement mechanisms, denoted as *EH_all_*, *EH*_2_, and *EH_w_*, have been considered.

- *EH_all_* only uses data for which all of the learning models made the same output. That is, data that all of the models output as “dangerous” is used as “dangerous” training data. Likewise, data that all of the models output “normal” is used as “normal” training data.
- *EH*_2_ follows the final decision of the above decision module *DM*_2_. That is, the data for which the final decision was “dangerous” is used as “dangerous” training data, and the data for which the final decision was “normal” is used as “normal” training data.
- *EH_w_* assigns different weights according to the decisions of the three models. It is the same as *EH*_2_, except that the data that all of the models output as “dangerous” are doubly copied and used for training data. If all of the models decide that some data are “dangerous”, the risk dataset is then slightly more enhanced, because the amount of risk training data has increased.

The performance analysis results according to the enhancement mechanisms are described in detail in Section 4. Since the size of the general data compared to the risk data is so large, some of the general data that are randomly extracted from the general data are used as training data. The new training data are added to the existing training data. The risk detection enhancement works periodically by the learning module.

## 4. Simulated Performance 

In this section, we describe the simulated results of the proposed traffic risk detection model through real driving on Seoul city roadways and Gyeongbu expressway. First, we analyze the performance of the proposed headway distance estimation against three aspects: real-time processing performance, vehicle detection, and the accuracy of the distance estimation. Then, we analyze the accuracy of the risk detection according to the proposed decision models and the proposed enhancement methods.

### 4.1. The Performance of the Headway Distance Estimation

**Figure 11:**
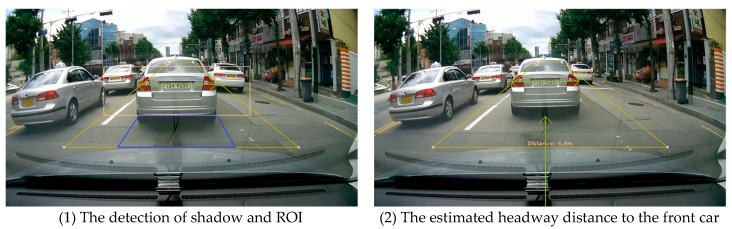
The implemented results of the proposed model.

Figure 11 shows the implemented result by our mobile application in real time. 

We have compared the throughput rate of image processing for three types of smart mobile devices. Table 1 shows the performance for the IPM image generation time, the shadow area detection time, the taillight detection time, and the number of image frames processed per second according to three different Samsung Galaxy series J7, A8, and S7. S7 showed the best performance; it took about 7 ms for the IMP image creation, and less than 0.1 ms for the shadow area detection, on all types of mobile devices. The taillight detection took the longest time; it took about 17 ms on all of the models. In addition, at least 37 image frames per second have been processed on all of the models. 

Secondly, we have analyzed the vehicle detection rate according to various driving environments. The driving environments have been classified into daytime urban roads, daytime expressways, and night in consideration of illuminance and speed. Table 2 summarizes the vehicle detection rate according to the driving environment. The target frame is defined as a frame that includes the shadow area of the front vehicle. The vehicle detection rate is measured with the number of frames where the detection of the front vehicle succeeded among the target frames. The detection rate during the daytime is more than 90%, and it is relatively higher on urban roadways. On the other hand, the detection rate at night was very low, less than 50%. As shown in Table 2, the shadow detection at night is still high, but the taillight detection rate is very low at night, since the taillight was not detected in red. Further research is needed to increase the taillight detection rate at night. 

Finally, the accuracy of the headway distance to the front vehicle has been analyzed. To achieve this, a laser-based distance-measuring sensor (LIDAR-Lite V3) has been additionally equipped to the experiment vehicle. In order to obtain more accurate data, two types of test vehicles (sedan and SUV) have been used in our experiment. The headway distance has been obtained by following the front experimental vehicle. The error between the laser sensor value and the estimated value of the proposed model has been analyzed. Table 3 shows the results of the error values for the two types of vehicles. As shown in Table 3, the distance accuracy is more than 96% within 15 m, but the accuracy decreases a little bit over long distances. The average error of the proposed model is 4.35%, whereas the average error of the laser sensor is 1.8%. However, the error rate is less than 5%, so we can conclude that it can be applied to the real situation practically. 

### 4.2. The Performance of the Traffic Risk Detection

Now, in this section, we describe the simulated results on the accuracy of the proposed traffic risk detection model in detail. We explain the experimental dataset first, and analyze the accuracy according to the proposed decision models and the enhancement methods.

#### 4.2.1. Experimental Data

The training data and test data are given from the actual driving data generated by two drivers. They commuted on Seoul city roads and the Gyeongbu expressway to collect these data. As mentioned before, we have collected the real dangerous deceleration data that both drivers empirically felt to be dangerous. Initially, 40 actual dangerous data were obtained, and 20 experimental data that had similar patterns to the real dangerous data were added. Among them, 26 dangerous training data were used to build up the initial learning module of our system, and the remainders were used as test data. Based on the initial learning module, our system has continuously collected new training and test datasets by detecting dangerous situations during actual driving. The Samsung Galaxy S7 smart phone was used in this experiment. For each deceleration segment, it takes 0.2 s until the final decision. The newly obtained data have been used as training data to reinforce the learning module, or have been used as test data. Two types of test datasets have been used. Type_1 test data are similar to the training data, but Type_2 test data include certain risk situations that are not similar to the training set. Table 4 shows the size of the experimental data set used in the simulation.

As mentioned above, the new input data with the final decision confirmed by our system is recycled as training data to reinforce the learning model. Table 5 shows the training data used for the three types of reinforcement algorithms. Three steps of enhancement have been performed in total, and the table shows the datasets used for each. 

#### 4.2.2. The Accuracy of the Traffic Risk Detection Model

We have defined two types of scores to analyze the accuracy of our risk detection model. The first indicator is a risk score denoted as *RScore*, which measures only the accuracy of the risk data. Thus, it shows how well our model detects a danger situation. For each deceleration segment *DS*_i_, suppose that *class*(*DS*_i_) denotes the given label of *DS*_i_, and *predict*(*DS*_i_) means the label estimated by our model. Therefore, for all of the deceleration segment *DS* values, the *RScore* is defined as follows:

(15) RScore=Prob(predict(DS)==“risk”|class(DS)==“risk”)

The second indicator is a total score, which is denoted as *TScore*. *TScore* measures the accuracy of the proposed model for both general and risk data. *TScore* is defined as follows:

(16) TScore=Prob(predict(DS)==class(DS))

Consequently, we analyze the scores of the two proposed decision models for initial training data and the two types of test data, and compare the performance with existing learning models. We also compare the performances of the proposed three types of enhancement models. Since the simulated results are slightly different every time the learning models work with the given training data, we have analyzed the average results of 10 different experiments. 

Table 6 shows both the *RScore* and *TScore* of the initial risk detection model for the initial training data. Among the existing learning models, the random forest model shows the best accuracy for the training data. Among the proposed decision models, *DM*_2_ is more accurate than *DM_all_*, and *DM*_2_ shows the next best in the accuracy for the training data. Table 7 summarizes the performance of the initial risk detection model for the two types of test data. Among the existing learning models, the neural network model shows the best accuracy for the test data, in which *DM*_2_ is still shown to be superior to *DM_all_*. Therefore, *DM*_2_ offers the best overall performance.

As shown in the above experimental results, each learning model shows a different performance depending on the situation. While random forest has the best performance for the training data, it has the lowest performance for the test data used in our experiments. The neural network has excellent performance for the test data rather than the training data. *k*NN shows moderate performance across the training and test data. We believe that it is appropriate to use all three models, because all types of sudden braking, whether those are similar to the training data or not, can occur in the actual situation.

Next, we describe the performance analysis results according to various enhancement methods. Each learning model has been updated by three enhancement techniques, respectively. Table 8 summarizes the *RScore* of each updated risk detection model for its new training data. The random forest and *k*NN show better accuracy for the training data, even in the enhanced models, than the neural network model. DM_2_ offers the next-best performance in the enhanced models as well. The *TScore* of the updated risk detection model for its new training data is more than 0.99 for all of the cases. 

**Figure 12:**
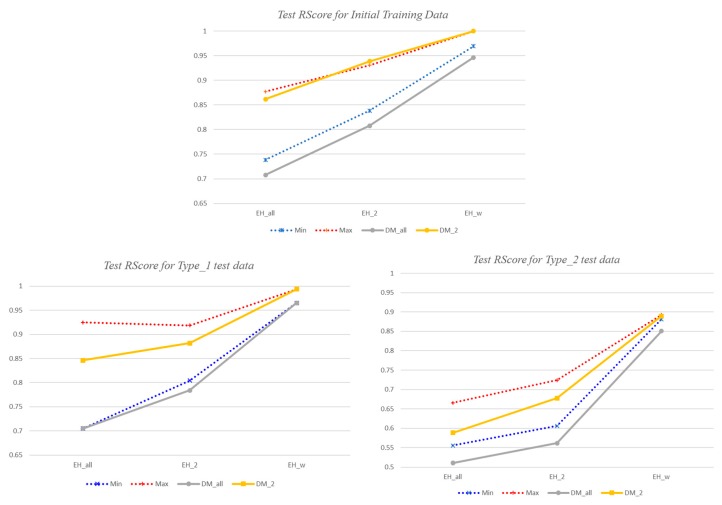
*RScore* of the enhanced models for the initial training data and test data.

Figure 12 shows the *RScore* of each risk detection model updated by each enhancement method for two types of test data. Since the three learning modules produce different performances depending on the enhancement techniques and the types of test data, it shows the *RScore* of our proposed two decision models compared with the minimum and maximum accuracy values produced by the three learning modules. As shown in Figure 12, the *RScore* of *EH_w_* is the best, as well as the same as the maximum value among the *RScores* of the three learning modules for all kinds of test data. In contrast, the *RScore* of *EH_all_* is the worst. In the *EH_all_* method, the risk detection model is updated with only data that all of the learning modules deemed to be a “risk”. That is, only limited learning data on risk situations are used for the risk detection reinforcement. Therefore, the detection rate is inevitably low. In both *EH*_2_ and *EH_w_*, since all of the data that at least two learning modules have judged as a “risk” are used to reinforce the risk situation, more diverse risk situations are reflected to the updated model. Furthermore, in *EH_w_*, if all of the models decide that the data is a “risk”, the data is duplicated and used as new training data, so that the training data for the risk situation increases, and *EH_w_* has a higher accuracy for the detection of dangerous situations than *EH*_2_.

According to the experimental results, the initial *DM*_2_ model has an *RScore* of 0.95 for Type_1 test data, but the *RScore* of the *DM*_2_ model enhanced by *EH_w_* is 0.99, which is close to the accuracy of 1. The *RScore* for the initial training data has been improved to 1 as well. The initial *DM*_2_ model has an *RScore* of about 0.71 for Type_2 test data, while the *RScore* of the *DM*_2_ model enhanced with *EH_w_* is 0.89. Thus, the accuracy increased by about 0.18 points. This score is still unsatisfactory, but it is expected to further improve as learning data on risk situations is continuously added. As a result, we can conclude that our proposed enhancing module increases the detection rate for various dangerous situations, including the initial training data.

**Figure 13:**
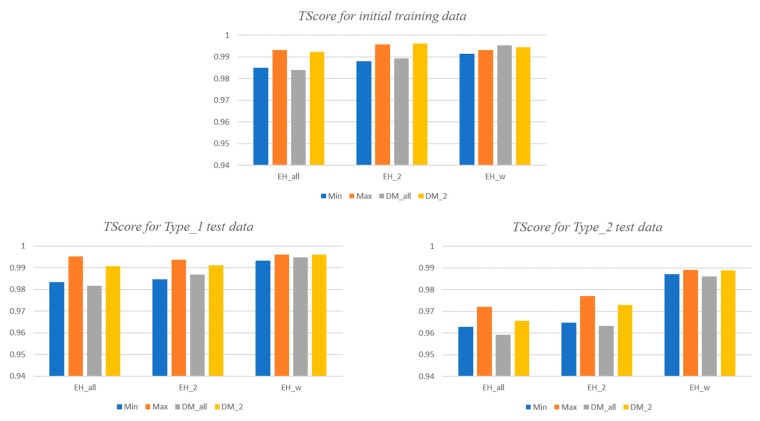
*TScore* of the enhanced models for initial training data and test data.

Figure 13 shows the *TScore* of the enhanced models for two types of test data, including the initial training data. The *TScore* indicates the accuracy of both the risk detection rate and the general situation detection rate. For the initial training data and the Type_1 test data, every type of enhanced model shows a similar accuracy of more than 0.98. For Type_2 test data, only models enhanced with *EH_w_* still show an accuracy of 0.98 or better, while the remainder is somewhat lower. It is determined that the low *RScore* for Type_2 also affects the *TScore*. Nevertheless, the *TScore* is 0.96 or more for all of the enhancement models, and the *TScore* deviation according to enhancement techniques is less than 0.02. Therefore, the performances of all of the reinforcement models are similar in terms of *TScore*.

As a result, we can conclude that the *DM*_2_ decision model enhanced with *EH_w_* shows the best performance for risk situation detection from our experiments. Risk situations occur very rarely under normal driving conditions, so there is overwhelmingly more data on general situations than risk situations, even if some selected general situations are used for learning. Therefore, *EH_w_*, which can train more dangerous situations, even though the data used is redundant, is more suitable for detecting dangerous situations than *EH*_2_.

## 5. Conclusions and Future Work

We have proposed a real-time traffic risk detection model based on a smart mobile device. Our model monitors driving behaviors using only the sensors of a smart mobile device, and extracts deceleration segments. We have also proposed a practical vehicle detection and headway distance estimation scheme for risk detection. Since emergent braking happens almost inevitably and exclusively in risk situations, our model detects risk situations by analyzing deceleration patterns using machine learning algorithms. We have used actual driving data obtained from two drivers by driving on Seoul city roadways and the Gyengbu expressway for three months in order to develop normal and dangerous driving deceleration patterns. Three different machine learning algorithms, which are neural network, random forest, and clustering, were used in parallel to build our learning module. We have also suggested two decision algorithms and three enhancement algorithms to improve our risk detection model continuously. Finally, we have analyzed the performance of the headway distance estimation and the risk detection accuracy of our proposed decision models and enhancing models. From our simulated results, the vehicle detection rate on urban roads at daytime is more than 92%, and the detection rate on highways is still more than 90%. The accuracy of the headway distance estimation is more than 96% within 15 m. The traffic risk detection model applied with decision model *DM*_2_ and enhanced with *EH_w_* shows the best performance on both the training data and test data. The model shows an accuracy of 0.99 for risk situations such as the training data, and shows a detection accuracy of 0.89 for unfamiliar risk situations. 

We need further research to improve the detection rate at night. In addition, we plan to extend our work to a model that can reflect not only deceleration patterns but also various driving behaviors, such as steering, handling, and emergent acceleration for risk situation detection.

## Figures and Tables

**Table 1:** Average image processing throughput according to smart mobile devices.

Items	Samsung Galaxy Mobile Devices		
	J7	A8	S7
IPM image creation time	7.633 ms	7.278 ms	6.862 ms
Shadow area detection time	<0.1 ms	<0.1 ms	<0.1 ms
Taillight detection time	17.895 ms	17.261 ms	16.344 ms
The number of image frames per second	37 frames	38 frames	41 frames

**Table 2:** The vehicle detection rate according to the driving environment.

Items	Daytime Urban Roads	Daytime Expressway	Night
The number of total frames	1440	1440	1440
The number of target frames	1145	1068	1180
The number of detected frames	1060	963	588
Detection rate (%)	92.58	90.17	49.83

**Table 3:** The result of following distance estimation.

Real Distance	Types of Vehicle	
	Sedan (Avante)	SUV (Tuscan)
10 m	10.23 m	10.00 m
15 m	15.51 m	14.82 m
20 m	21.47 m	19.16 m
The average error rate	4.35%	1.80%

**Table 4:** Experimental dataset.

	Initial Training Data	Test Data Type_1	Test Data Type_2
Danger	26	29	74
Normal	445	443	796
Total	471	472	870

**Table 5:** New training dataset for enhancing the learning module.

	*EH_all_*			*EH*_2_			*EH_w_*		
	Danger	Normal	Total	Danger	Normal	Total	Danger	Normal	Total
Step 1	41	937	978	54	935	989	51	952	1003
Step 2	63	1507	1570	75	1519	1594	110	1514	1624
Step 3	77	2085	2162	99	2101	2200	160	2086	2246

**Table 6:** The accuracy of the initial risk detection model for the initial training data. *k*NN: *k* Nearest Neighbors.

*k*NN		Random Forest		Neural Network		*DM_all_*		*DM*_2_	
*RScore*	*TScore*	*RScore*	*TScore*	*RScore*	*TScore*	*RScore*	*TScore*	*RScore*	*TScore*
0.8923	0.9940	0.9846	0.9991	0.9231	0.9957	0.8923	0.9940	0.9231	0.9957

**Table 7:** The accuracy of the initial risk detection model for two types of test data.

	*k*NN		Random Forest		Neural Network		*DM_all_*		*DM*_2_	
	*RScore*	*TScore*	*RScore*	*TScore*	*RScore*	*TScore*	*RScore*	*TScore*	*RScore*	*TScore*
**Type_1**	0.9247	0.9953	0.7708	0.9847	0.9522	0.9970	0.7229	0.9830	0.9593	0.9974
**Type_2**	0.7184	0.9765	0.5662	0.9639	0.7321	0.9777	0.5218	0.9601	0.7074	0.9756

**Table 8:** The *RScore* of each enhanced risk detection model for its new training data.

	*k*NN	Random Forest	Neural Network	*DM_all_*	*DM*_2_
*EH_all_*	0.986832612	0.974025974	0.950108225	0.93953824	0.974025974
*EH*_2_	0.956668	0.974108	0.910759	0.892948	0.952627
*EH_w_*	0.968773006	0.99125	0.934026074	0.914026074	0.982523006

## References

[1] Kaur R., Sobti R. Current Challenges in Modelling Advanced Driver Assistance Systems: Future Trends and Advancements. Proceedings of the IEEE International Conference on Intelligent Transportation Engineering.

[2] iOnRoad iOnRoad Augmented Driving Pro. https://ionroad-pro.en.aptoide.com/.

[3] Movon Corporation Movon FCW. https://apkpure.com/movon-fcw/com.movon.fcw.

[4] Danescu R., Itu R., Petrovai A. (2016). Generic Dynamic Environment Perception Using Smart Mobile Devices. Sensors.

[5] Mohan P., Padmanabhan V.M., Ramjee R. Nericell: Rich Monitoring of Road and Traffic Conditions Using Mobile Smartphones. Proceedings of the ACM Conference on Embedded Network Sensor Systems.

[6] Kalra N., Chugh G., Bansal D. (2014). Analyzing Driving and Road Events via Smartphone. Int. J. Comput. Appl..

[7] Gozick M.B., Dantu R., Bhukhiya M., Gonzalez M.C. (2012). Safe Driving Using Mobile Phones. IEEE Trans. Intell. Transp. Syst..

[8] Fischer R.W., Fischer J.R., Ricaurte W.A. (2018). Safe Driving Monitoring System. U.S. Patent.

[9] Albertus G., Meiring M., Myburgh H.C. (2015). A Review of Intelligent Driving Style Analysis Systems and Related Artificial Intelligence Algorithms. Sensors.

[10] Ly M.V., Martin S., Trivedi M.M. Driver Classification and Driving Style Recognition using Inertial Sensors. Proceedings of the IEEE Intelligent Vehicles Symposium (IV).

[11] Lavie S., Jacobs F., Fuchs G., Bergh J.V.D. (2018). System and Method for Use of Pattern Recognition in Assessing or Monitoring Vehicle Status or Operator Driving Behavior. U.S. Patent.

[12] Deng C., Wu C., Lyu M., Huang Z. (2017). Driving style recognition method using braking characteristics based on hidden Markov model. PLoS ONE.

[13] Xiao J., Liu J. Early Forecast and Recognition of the Driver Emergency Braking Behavior. Proceedings of the International Conference on Computational Intelligence and Security.

[14] Shin M.J., Oh K.J., Park H.M. (2015). Accident Alert System for Preventing Secondary Collision. U.S. Patent.

[15] Lee J., Park S., Yoo J. (2016). A Location-based Highway Safety System using Smart Mobile Devices. J. KIISE.

[16] WHO Global Status Report on Road Safety. http://www.who.int/violence_injury_prevention/road_safety_status/2015/en/.

[17] Tuohy S., O’Cualain D., Jones E., Glavin M. Distance Determination for an Automobile Environment Using Inverse Perspective Mapping in OpenCV. Proceedings of the 2010 IET Signal and Systems Conference (ISSC).

[18] Sathyanarayana A., Sadjadi S.O., Hansen J.H.L. Leveraging Sensor Information from Portable Devices towards Automatic Driving Maneuver Recognition. Proceedings of the International IEEE Conference on Intelligent Transportation Systems.

[19] Breiman L. (2001). Random forests. Mach. Learn..

